# Functional properties of bone marrow derived multipotent mesenchymal stromal cells are altered in heart failure patients, and could be corrected by adjustment of expansion strategies

**DOI:** 10.18632/aging.100716

**Published:** 2015-01-09

**Authors:** Renata I. Dmitrieva, Alla V. Revittser, Maria A. Klukina, Yuri V. Sviryaev, Ludmila S. Korostovtseva, Anna A. Kostareva, Andrey Yu. Zaritskey, Evgeny V. Shlyakhto

**Affiliations:** ^1^ Federal Almazov Medical Research Centre, St. Petersburg, Russia; ^2^ St. Petersburg State Polytechnical University, Branch of Medical Physics and Bioengineering, Russia

**Keywords:** heart failure, bone marrow multipotent mesenchymal stromal cells, proliferation, differentiation, myofibroblas, natriuretic peptides system, senescence, hypoxia, cell therapy

## Abstract

Background: Bone marrow multipotent mesenchymal stromal cells (BM-MMSC) considered as a prospective substrate for cell therapy applications, however adult stem cells could be affected by donor-specific factors: age, gender, medical history. Our aim was to investigate how HF affects the functional properties of BM-MMSC. Materials and methods: BM-MMSC from 10 healthy donors (HD), and 16 donors with chronic HF were evaluated for proliferative activity, ability to differentiate, replicative senescence, expression of genes that affect regeneration and fibrosis. The effect of culturing conditions on efficiency of BM-MMSC expansion was determined. Results: HF-derived BM-MMSC demonstrated early decrease of proliferative activity and upregulation of genes that control both, regeneration and fibrosis: Tgf-β pathway, synthesis of ECM, remodeling enzymes, adhesion molecules. We assume that these effects were related to increase of frequency of myofibroblast-like CD146+/SMAα+ CFU-F in HF samples; (ii) low seeding density and hypoxia resulted in predominant purification and expansion of CD146+/SMAα- CFU-Fs. (iii) the activity of NPs system was downregulated in HF BM-MMSC; Conclusions: downregulation of NP signaling in combination with upregulation of Tgf-β pathway in BM-MMSC would result in pro-fibrotic phenotype and make these cells non-effective for therapeutic applications; the corrections in culturing strategy resulted in 2^3^-2^7^ increase of expansion efficiency.

## INTRODUCTION

Chronic heart failure (HF) is one of the most common causes of death worldwide, and the only radical treatment for severe chronic HF remains to be heart transplantation. However, in many cases the transplantation is not a solution due to the limited availability of donors, high cost and problems associated with immunosuppression [1]. Therefore, it is necessary to search for new therapeutic approaches to restore the structure and function of cardiac muscle. In the past two decades cell therapy has been considered as the prospective therapeutic approach to the treatment of cardiovascular diseases including HF [1-9].

The cells intended for cell therapy must have certain characteristics: they should be relatively easy available, safe, and demonstrate efficiency in stimulation of reparation of cardiac muscle [8]. Different cell types were tested in regeneration protocols: multipotent mesenchymal stromal cells from bone marrow and adipose tissue, bone marrow mononuclear cells, skeletal muscle myoblasts, fetal cardiomyocytes, fibroblasts, and others [2, 8, 10-12]. By now BM-MMSC remain to be the most attractive, and one of the best characterized substrates for clinical applications: these cells could be rapidly and efficiently expanded in vitro and this type of cells is known to be immunologically privileged. The latter makes possible to consider BM-MMSC for allogeneic transplantation [13-17]. Though there are clinical evidence that the low immunogenicity of MMSC enables the use of allogeneic MMSC transplantation [18], many researchers still believe that the ideal substrate for cell therapy are autologous cells [7, 8]. However, it was demonstrated in several animal-based studies that donor-specific factors could attenuate stem cell functions and reduce regenerative potential [19][20]. Influence of donor's age and gender on the properties of BM-MMSC has been studied actively in recent years in many laboratories [19-24], but the studies of impact of chronic cardiovascular disorders, including HF, on multipotent progenitor cells are limited [24-27]. In the present work the direct impact of HF on functional properties of BM-MMSC was estimated, and the possibilities to compensate for these effects by correcting ex vivo expansion strategies of BM-MMSC were studied.

## RESULTS

### BM-MMSC characterization

**Figure 1:**
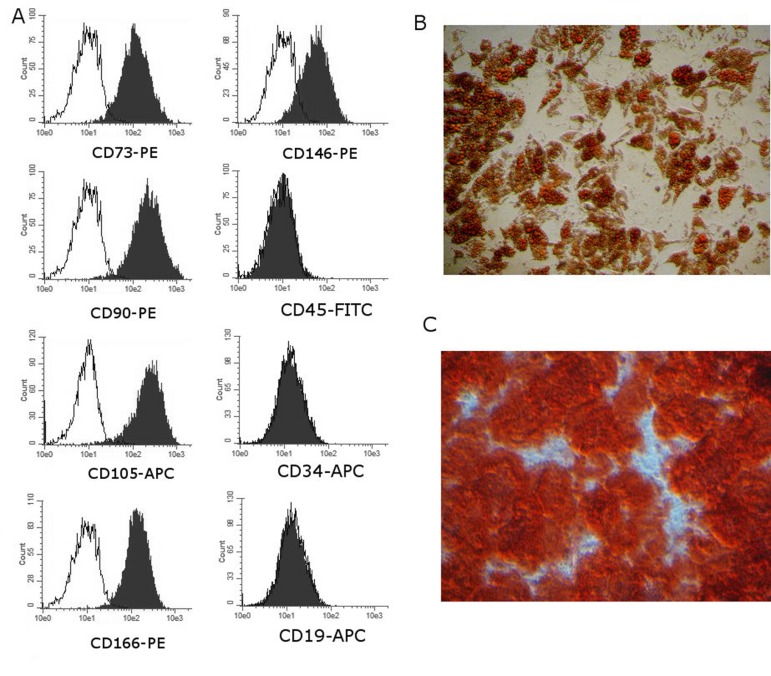
Representative results of routine analysis of BM-MMSC samples quality (**A**) Immunophenotype; Performed on Guava EasyCyte 8 using ExpressPro software. (**B,C**) Ability to differentiate: (**B**) Adipogenic differentiation (x200, OilRedO staining) and (**C**) osteogenic differentiation (x200, Alizarin staining).

All cells used in this project were analyzed routinely for expression of stromal cell-associated markers CD105, CD90, CD166 and CD73 and for hematopoietic lineage cells markers CD34, CD19, CD45 to make sure that cells meet minimal criteria for defining multipotent mesenchymal stromal cells [12]. All BM-MMSC samples were CD105/CD90/CD166/CD73 positive and negative for CD34/CD19/CD45 at all tested passages. Additionally, the ability of BM-MMSC to differentiate to osteo- and adipo-lineages was tested. Representative images are demonstrated on Figure 1.

### BM-MMSC proliferative activity

**Figure 2:**
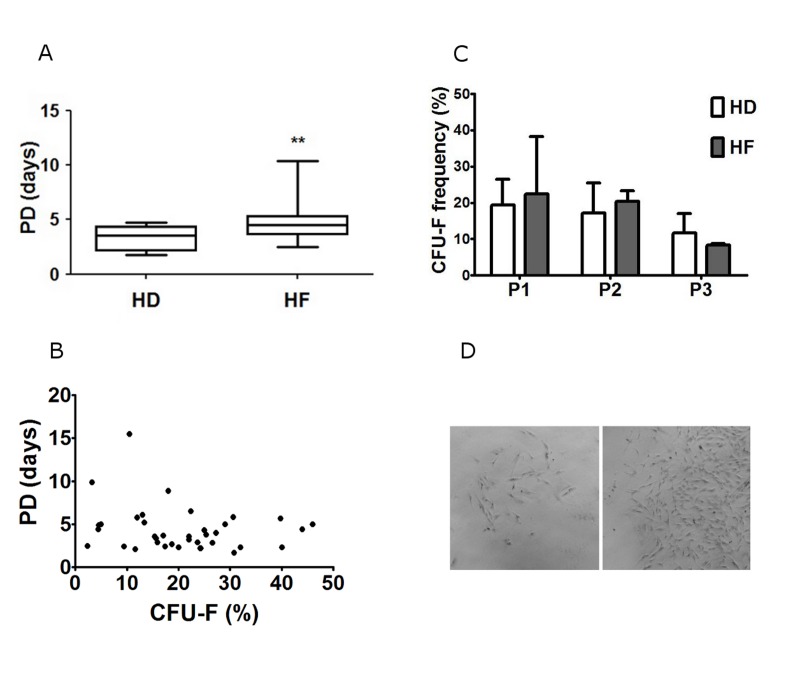
Analysis of in vitro proliferation activity of HD and HF BM-MMSC cells seeded at 3000 cells/cm2 and expanded in normoxia (pO2 =20%) (**A**) The population doubling time (PD) at passage 3; Data are presented as mean +/− SEM; n=16 for HF, and n=10 for HD; ** p <0.03; (**B**) CFU-F frequency in BM-MMSC samples at successive passages; Data are presented as mean +/− SEM; n=5. (**C**) The correlation analysis between CFU-F frequency and PD in BM-MMSC samples; (**D**) BM-MMSC CFU with low (left panel) and high (right panel) proliferation rate; (x50).

Proliferative activity is an important feature of cell samples that intended for therapeutic protocols. The decline in proliferative activity could indicate that cells either enter the state of replicative senescence [28, 29] or they just stop growing and begin to differentiate [30]. To estimate the proliferative activity in BM-MMSC we calculated population doubling time (PD) for each sample in successive passages, and found out that at the first steps of in vitro expansion PD was less than 24 h in practically all tested samples, but increased gradually during expansion, and could reach more than 7 days by P3. The significant difference in proliferative activity between HD and HF samples was detected after about 14 in vitro doublings (Figure 2A).

### Dynamics of CFU-F frequency during expansion

**Figure 3:**
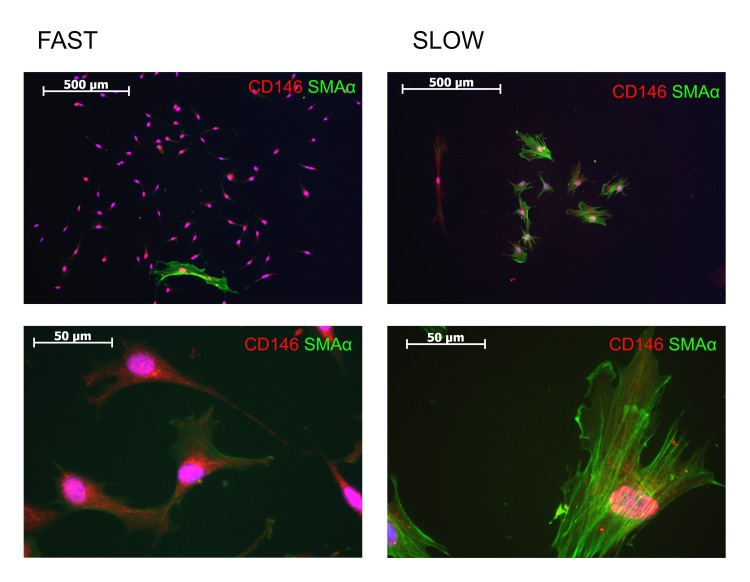
Analysis of expression of CD146 and SMAα in “slow” and “fast” CFU-Fs

The frequency of CFU-F in sample is another common indicator of BM-MMSC proliferative activity. Surprisingly, we did not find the significant difference in CFU-F frequency between HD and HF (Figure 2B). Furthermore, the correlation between CFU-F and PD was not observed in our samples (Figure 2C), which means that slow and fast proliferating BM-MMSC samples differ not in frequency of CFU-Fs, but rather in functional properties of CFU-Fs. Indeed, it was clear, that the properties of different CFU-Fs in every particular sample were not identical: in all samples we were able to observe two different kinds of colonies: small, slow proliferating, and large, rapidly proliferating CFU-Fs. “Fast” colonies demonstrated high proliferation rate and high cellular density (small distance between cells in colony, that indicates low migratory capacity). Cells in “slow” colonies had large nuclei, were larger in size, than in “fast” colonies, did not proliferate actively, and demonstrated higher distance between cells, indicating increase in migratory activity (Figure 2D). In both types of colonies cells were positive for CD146 (Figure 3), the adhesion molecule which is considered as one of putative markers of multipotensy in mesenchymal stromal cells [28, 31]. Additionally, cells in “slow” colonies were positive for quite well-organized SMAα filaments, which suggest initiation of BM-MMSC-to-myofibroblasts differentiation process (Figure 3).

Unfortunately, the method we used here to estimate the CFU-F frequency in BM-MMSC samples does not allow distinguish between “slow” and “fast” CFU-Fs. However, since the decrease in PD was not accompanied by decrease in frequency of total (“slow” plus “fast”) CFU-Fs in HF-derived samples, we hypothesized that the decrease in proliferation rate in HF-derived BM-MMSC is an indicator of increased frequency of slow proliferating, SMAα+ myofibroblast-like CFU-Fs. The next part of our study was performed to test this assumption.

### Remodeling pathways are activated in BM-MMSC from HF patients

The differentiation of stromal cells/fibroblasts to myofibroblasts is an initial step in regulation of wound healing and tissue regeneration. These myofibroblasts proliferate in response to injury, express SMAα, secrete and process extracellular matrix (ECM) proteins to form a scar or fibrosis [32-36]. We hypothesized that BM-MMSCs derived from HF patients were activated in vivo to differentiate into myofibroblast/myofibroblast-like cells in order to accelerate regeneration program, and this commitment affected proliferative activity of BM-MMSC during expansion in vitro. To test this hypothesis we compared the expression of key regulators of tissue remodeling and regeneration in HD and HF-derived BM-MMSC.

**Figure 4:**
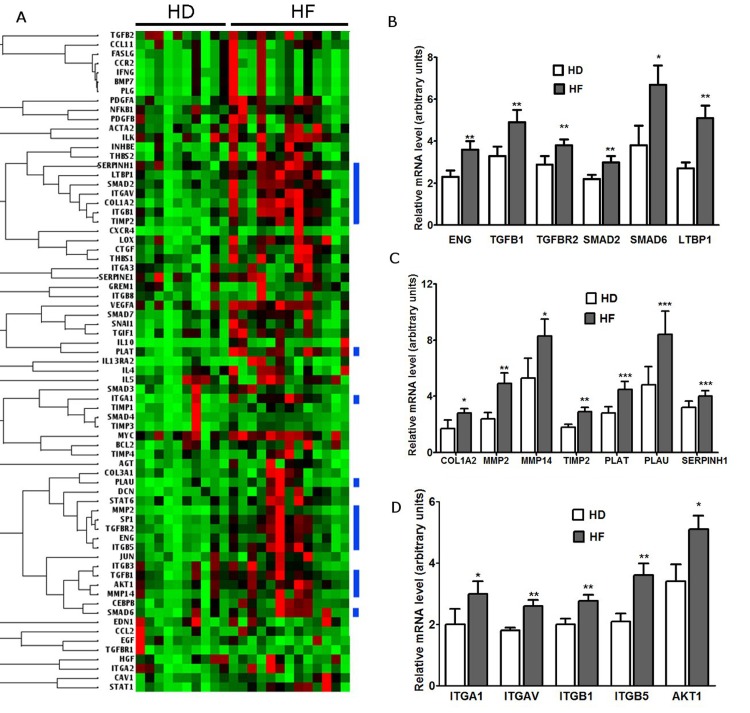
Analysis of expression of genes that regulate regeneration and remodeling in BM-MMSC from HD and HF patients (**A**) Visualization of analysis of RT2-PCR expression array results: the blue color bars represent clusters of upregulated genes that control tissue regeneration at different levels; (**B**) Validation of upregulation of Tgf-beta superfamily genes in HF-derived BM-MMSC; n=10-16, *p<0.02, **p<0.01; (**C**) Validation of upregulation of extracellular matrix components and remodeling enzymes in HF-derived BM-MMSC; n=10-16; *p<0.001, **p<0.01, ***p<0.05; (**D**) Validation upregulation of cellular adhesion molecules and regulators of epithelial-to-mesenchymal transition in HF-derived BM-MMSC; n=10-16; *p<0.02; **p<0.002. All data are presented as mean +/− SEM.

First, the screening of the expression of 84 key genes involved in tissue remodeling during the repair and healing was done using RT^2^ Profiler PCR. The panel included genes that regulate different stages of both, tissue regeneration and fibrosis: inflammation, proliferation and remodeling. The results of screening revealed that virtually all genes presented on panel were upregulated in HF-derived samples (Figure 4A). Afterwards, the RT-PCR analysis of expression of individual genes was performed to verify RT^2^ Profiler results. All genes selected for further analysis fall into three functional groups: Tgf-beta superfamily genes; extracellular matrix components and remodeling enzymes; cellular adhesion molecules and regulators of epithelial-to-mesenchymal transition. The results of analysis are presented on Figure 4 (B-D): these results confirm our hypothesis that in HF-derived BM-MMSC samples the signaling pathways that regulate both, tissue repair and remodeling were activated at least at mRNA level, and provide a valuable background information for planning further research aimed to reveal the molecular networks that control BM-MMSC response/adaptation to chronic HF.

### The regulation of natriuretic peptide signaling system is altered in HF derived BM-MMSC

It is obvious that functional alterations in bone marrow cells derived from HF patients should be determined by systemic disease-specific mechanisms. HF is characterized by chronic activation of natriuretic peptides system: the secretion and circulation of cardiac natriuretic peptides (NPs) are markedly increased during HF. NPs system not only regulates diuresis, natriuresis and vasorelaxation, but also is essential for maintenance of normal cardiac architecture [37, 38].

**Figure 5:**
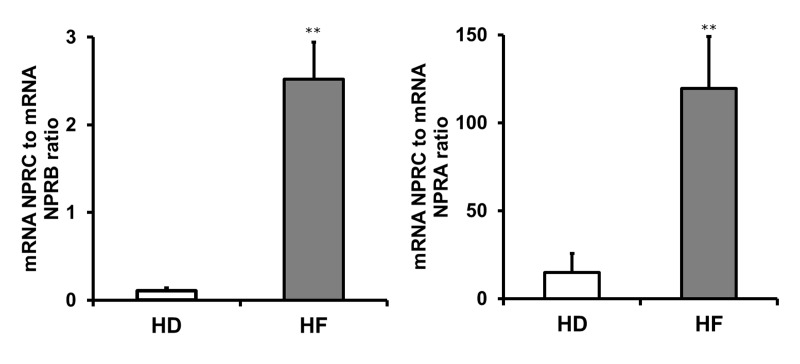
The expression of regulatory receptors (NPRA, NPRB) of natriuretic peptides system is downregulated in HF-derived BM-MMSC n=4-5; **p<0.02; Data presented as mean +/− SEM. NPRC is used as a reference gene.

Furthermore, NPs receptors modulate cardiomyocyte proliferation during development [39], and participate in regulation of cardiac remodeling and regeneration [40-43]. We hypothesized, that the changes in regeneration-related functional properties of BM-MMSC in HF could be, at least in a part, the result of alterations in regulation of NPs system. The activity of NPs system is determined by the reciprocal control of clearance (NPRC) and signaling (NPRA/NPRB) receptors [44-46], therefore we tested if there are alterations in regulation of NPRC, NPRA and NPRB receptors in HF-derived BM-MMSC. The results are demonstrated on Figure 5: the data are presented as the ratio of clearance receptor NPRC mRNA level to regulatory receptor (NPRA, NPRB) mRNA level. The downregulation of NPs system at mRNA level was demonstrated for HF-derived BM-MMSC, and these results are in a good accordance with previous data showing on experimental animal models that NP receptors could be downregulated in failing heart by high levels of their agonists [47-49].

### Culturing strategy affects the efficiency of BM-MMSC in vitro expansion

The early decrease in proliferative activity would be a serious obstacle in development of cell-based therapeutic protocols, therefore the next step in our study was aimed to estimate how to increase the efficiency of HF-derived BM-MMSC sample expansion in vitro by modifying the culturing strategy. The experimental approach was based on the assumption that each sample, including those with low proliferation rate, would still contain a fraction of “fast” colonies, and purification and expansion of this particular fraction would result in stabilization of proliferation activity of BM-MMSC sample in vitro. The true clonogenic/stem cells are capable of forming colonies of fibroblast-like cells at very low plating densities, and cell contacts would even inhibit cells growth. On the contrary, non-clonogenic/activated cells would need cell-to-cell contacts to survive [28]. Thus, we tested the possibility to increase the efficiency of expansion of BM-MMSC by reducing the seeding density from 3000 to 100 cells/cm2 in order to provide the room for expansion of rapidly proliferating colonies and minimize the contact inhibition of growth [28]. Additionally, the effect of moderate hypoxia (pO2=5% instead of pO2=20%) on expansion efficiency was tested as described earlier [50].

**Figure 6:**
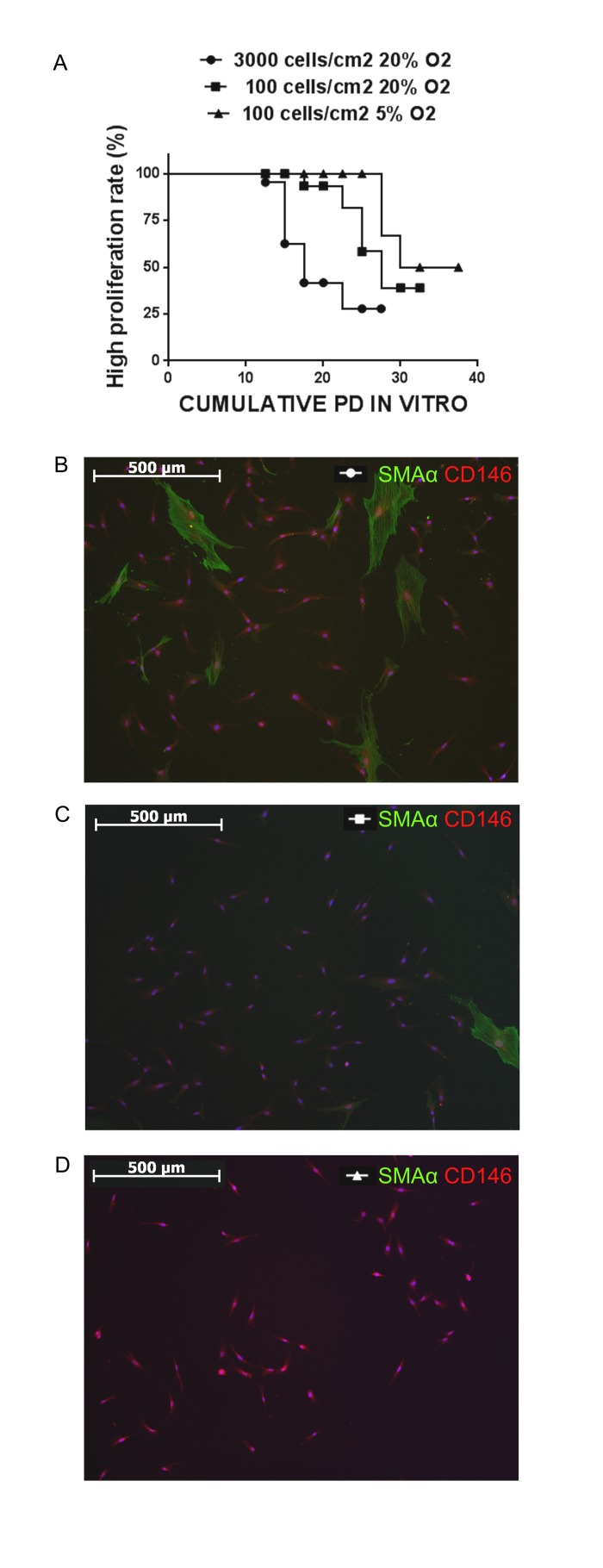
Culturing strategy affects the phenotype of cellular population and the efficiency of in vitro expansion (**A**) Kaplan-Meier survival curves for analysis of proliferation rate during BM-MMSC expansion in vitro under different culture conditions. The log-rank tests confirmed the significant difference for each pair of culturing conditions (p<0,001). (∉) n=43; (■) n=20; (▲) n=21. (**B**-**D**) The frequency of SMAα+ cells in cultures seeded at low density in both, normoxia and hypoxia is reduced compared to cultures seeded high density.

The results of this set of experiments shown in Figure 6A: cells plated at seeding density 3000 cells/cm2 under pO2=20% maintained high proliferative rate with no signs of replicative senescence only for up to 17,5 cumulative population doublings (median survival), while at seeding density 100 cells/cm2 under pO2=20% the median survival was 27,5 population doublings, and increased further up to 33,75 when cells were seeded at low density and cultured under moderate hypoxic conditions (pO2= 5%). Furthermore, the frequency of SMAα+ cells in cultures seeded at low density in both, normoxia and hypoxia, was noticeably reduced compared to cultures seeded high density (Figure 6 B-D).

## DISCUSSION

In present work we have found that a number of properties were altered in BM-MMSC derived from HF patients compared to HD-derived BM-MMSC. In particular, in HF-derived BM-MMSC the decrease in proliferative activity during *in vitro* expansion was detected, accompanied by upregulation of signaling pathways that control both, tissue regeneration and fibrosis. We assume that decline in proliferative activity was related to increase of frequency of “slow” myofibroblast-like CD146+/SMAα+ CFU-F in HF-derived samples. The switch to myofibroblast-like phenotype in HF-derived BM-MMSC indicates that cells were committed to enter disease-induced remodeling program. This assumption supported by the results presented in Figure 4: in HF samples we have detected the significant upregulation of signaling pathways that regulate tissue remodeling, scar formation and maturation, synthesis of ECM components, remodeling enzymes and adhesion molecules. However, the switch to myofibroblast-like phenotype and upregulation of regeneration pathways in cultured HF-derived BM-MMSC could not be unequivocally considered as a confirmation of better therapeutic potential of these cells, since common molecular mechanisms trigger both, the reparative and pathological aspects of regeneration and fibrosis, and inappropriate activation of subsequent steps in complex and coordinated process of wound healing would result in pathological functional impairment in injured tissue [33]. For instance, Tgf-β signaling pathway plays important role in both wound healing and fibrosis in multiple tissue types: in the early stages of wound healing Tgf-β promotes fibroblast to myofibroblast conversion and directly upregulates collagen synthesis via activation of the Smad signaling pathways [33, 51]. Because of that, it is important to identify tissue- and disease-specific mechanisms that selectively promote the regenerative/anti-fibrotic activity of “bi-potential” signaling pathways, and learn how to control the activity of this signaling. In this regard we decided to test if there are any alterations in regulation of NP system in HF-derived BM-MMSC. There were several reasons to suggest that NP system could be involved it regulation of anti-fibrotic/pro-fibrotic potential of BM-MMSC in HF: first, there are the evidence on literature that BNP has a direct effect on cardiac fibroblast to inhibit fibrotic responses and prevent cardiac remodeling in pathological conditions [52]; second, it was demonstrated by the number of independent studies that NP system plays a very important role in regulation of cardiac development [39], cardiac hypertrophy and remodeling [47, 48, 52, 53]. In our work we provide the first evidence that in HF-derived BM-MMSC the activity of NPs system is downregulated (Figure 5). We suggest that downregulation of NP signaling in combination with upregulation of TGf-β signaling in BM-MMSC would result in pro-fibrotic phenotype which could make these cells non-effective for therapeutic applications. Certainly, further investigations are required to reveal the molecular mechanisms that regulate interactions between reparatory, profibrotic and antifibroic pathways in HF-derived multipotent cells including BM-MMSC.

In the second part of our work we have demonstrated that decrease in efficiency of expansion could be markedly improved by culturing of BM-MMSC under moderate hypoxic conditions and substantial decrease in cell seeding density (Figure 6). In general, our data are in a good accordance with previous records indicating that the seeding density affects proliferative activity of the BM-MMSC sample [18, 54-56]. Furthermore, there are data, demonstrating that clonal seeding in combination with strong hypoxia (pO2=1%) results in increase of efficiency of MMSC expansion in vitro due to prevention of early onset of replicative senescence [54]. However, there is still no generally accepted opinion on molecular mechanisms that drive these effects. We have made an attempt to reveal some aspects of these mechanisms. We have mentioned above that the decline in proliferative activity could indicate that cells either enter the state of replicative senescence [28, 29] or they just stop growing and begin to differentiate [30]. Therefore, in order to achieve the stabilization of proliferative activity it is necessary to prevent the onset of cellular senescence and the spontaneous differentiation during expansion *in vitro*. In our experiments the reduction in seeding density resulted in increase of proliferative activity of BM-MMSC samples accompanied by considerable decrease in frequency of myofibroblast-like CD146+/SMAα+ cells in culture (Figure 6). We suggest that the low seeding density promotes predominantly the expansion of clonogenic CD146+/SMAα- (“fast”) SFU-Fs, while “slow” myofibroblast-like CD146+/SMAα+ cells do not proliferate at these conditions, and would even die since non-clonogenic/differentiated cells need cell-to-cell contacts to survive in vitro [28]. The role of further increase of efficiency of BM-MMSC expansion under moderate hypoxia in our culture system remains unclear and deserves additional detailed investigations. We hypothesize that several factors might contribute to this effect. First, moderate hypoxia could prevent spontaneous BM-MMSC-to-myofibroblast differentia-tion in our cultures, as it was demonstrated for rat skin and cardiac fibroblasts [59, 60]. Second, hypoxia can decelerate premature conversion to senescence via either down-regulation of p21 [54], or mTOR inhibition [61, 62]. It is interesting to note that all these factors could be in a good agreement. There are evidence that p21 is a key signaling mediator that regulates the oxygen-promoted fibroblasts to myofibroblasts differentiation [63, 64]. The role of mTOR in irreversible loss of replicative potential is well documented and discussed [61], and hypoxia-induced inhibition of mTOR may explain why hypoxia stimulates proliferative potential in BM-MMSC.

Conclusions: in present work we have found that HF-derived BM-MMSC demonstrate early decrease of proliferative activity and upregulation of genes that control both, regeneration and fibrosis. We assume that these effects are related to increase of frequency of myofibroblast-like CD146+/SMAα+ CFU-F in HF samples, but low seeding density in combination with hypoxia resulted in predominant purification and expansion of CD146+/SMAα- CFU-Fs and prevented the loss replicative potential. Further experiments are necessary to learn how to manipulate the culturing conditions in order to predict and, most importantly, control the balance between proliferation rate, replicative senescence, regenerative potential, pro-fibrotic and anti-fibrotic properties of cellular sample intended for experimental or therapeutic protocols.

## MATERIALS AND METHODS

### Ethics statement

The research was conducted according to the principles stated in the Declaration of Helsinki. The samples were collected under agreement of the Institutional Ethics Committee at Federal Almazov Medical Research Centre. All patients and donors entering the program agreed to and signed an institutional review board-approved statement of informed consent.

### Donors

BM-MMSCs were isolated from bone marrow aspirates of healthy donors and patients enrolled in the Studies Investigating Co-morbidities Aggravating Heart Failure (SICA-HF). A total of 10 healthy adult donors, 16 patients with chronic heart failure were enrolled. All patients were diagnosed as having CHF based on clinical signs of heart failure and echocardiographic or angiographic evidence of impaired left ventricular systolic function, and have a disease history of at least 12 months. Patients had no diabetes and no anti-diabetic treatment or history of diabetes. The clinical diagnosis of HF was evidenced by at least one of the following: (1) left ventricular ejection fraction ≤40%; (2) left atrial dimension >4.0 cm; or (3) N-terminal pro-B-type natriuretic peptide >47.3 pM/L. Biochemical, clinical and demographic data are presented in Table 1.

**Table 1:** Biochemical, clinical, and demographic data of the study population

	HEALTHY DONORS (n=10)	HEART FAILURE (n=16)
Age, years	42.7+3	54.4+4,5**
BMI, kg/m^2^	27.3+2	26+1,3
NYHA class	N/A	II - IV
NT-proBNP, pM/L	0.0	67+21**
proANP, nM/L	0.83+0.2	9.5+0.8*
Ejection fraction (%)	N/A	32+3
Glucose, mM/L	5.3+0.16	5.06+0.02
Insuline, pM/L	68+20	59.5+31
HbA1c(%)	N/A	5.9+0.2
Total cholesterol, mM/L	5.2+0.4	5.1+0.4

Values are presented as mean + SEM;

* p<0.001;

** p<0.05.

N/A – data not available

### Bone marrow derived cell cultures (BM-MMSC)

BM aspirates were obtained from the iliac crest or sternum. Standard BM-MMSC cultures were established from plastic adherent BM cell fractions as described elsewhere, with some modifications [29]. Density gradient was used in the isolation procedure to eliminate unwanted cell types that were present in the marrow aspirate. A portion of cells isolated from the density interface (bone marrow mononuclear cells, BMMC) was used in CFU assay to estimate a frequency of colony forming units (CFU, see below) in each sample. The remaining cells were suspended and plated immediately in T75 flasks in culture medium at a density of about 3000 cells/cm2 of surface area for expansion and culture. HSC and non-adherent cells were removed with changes in medium. This initial passage of the primary cell culture was referred as a passage 0 (P0). Experimental culturing conditions were 3000 and 100 cells/cm2 of surface area them hypoxic conditions (Multi incubator MCO-18M (SANYO, Japan), the concentration of CO2 and O2 to 5 %) and normoxia (the concentration of CO2 5 % and O2 20%).

### Calculation of the number of cumulative population doublings (PD) and the time of PD

The most convenient parameter for documentation of long-term culture is counting of the number of cell passages, however, the normalization of results only to passage numbers may lead to deceptive conclusions. In this respect, calculation of in vitro PD is more accurate [57, 58]. The initial MSC number in BMMC sample can only be estimated by accounting colony-forming unit (CFU) frequency based on the assumption that every colony has been derived from a single clonogenic cell. CFU in BMMC was estimated as described [28]. Briefly, cell suspension was serially diluted two-fold across the 6 columns of 96-well plates, resulting in columns containing from 20000 to 625 cells per well. After 10-14 days of culture the number of positive and negative wells was determined for each cell concentration and CFU frequency in initial BMMC or ASC population was calculated. Thereafter, cell number was determined at all passages and time of PD and number of cumulative PD was calculated.

### Limiting dilution assay for CFU-F determination

Determination of frequency of colony forming units (CFU-F) in BM-MMSC samples was performed at subsequent passages: cell suspension was serially diluted two-folds across the 8 columns of 96-well plates, resulting in columns containing from 50 to 0,39 cells per well. After 10 days of culture, the number of positive and negative wells was determined for each cell concentration and CFU-F frequency in BM-MMSC population was calculated as described [28].

### Senescence assay

Senescence-associated β-galactosi-dase activity was used as biomarker for assessing replicative senescence in MSC according to manufacturer instructions (Sigma, C S0030-1KT).

### Flow cytometry

Cells were analyzed for hematopoietic lineage cells markers CD34, CD19, CD45 and for stromal cell-associated markers CD105, CD90, CD73, CD166 and CD146 by using directly conjugated phycoerythrin (PE), fluorescein isothiocyanate (FITC) or allophycocyanin (APC) antibodies (Becton-Dickinson BioSciences, San Jose, CA, USA) on a Guava EasyCyte 8 using ExpressPro software.

### Stimulation of adipogenesis

Adipogenesis was induced by replacing the culture media with adipocyte induction medium composed of culture medium supplemented with 1μM of insulin, 1 μM of dexamethasone and 1 μM of IBMX. Cells were maintained in culture for up to 14 days, and adipocyte differentiation was determined by Oil Red staining after fixation in 4% PFA.

### Stimulation of Osteogenesis

Osteogenesis was induced by replacing the culture medium with osteogenic induction medium composed of culture medium supplemented with 10 mM β-glycerophosphate, 10 nM dexamethasone, 50 μg/ml sodium ascorbate 2-phosphate. Cultures were maintained in culture for 21 days. Then cultures were rinsed in PBS, fixed in 70% ethanol, and osteogenic differentiation was determined by staining for calcium phosphate with Alizarin red.

### RNA isolation and real-time PCR analysis

Total RNA was isolated using Aurum Total RNA Mini Kit (Bio-Rad, cat #732-6820). RNA quality was verified using RNA LabChip Kit (Agilent, RNA 6000 Nano) and concentration measured with a NanoDrop ND-1000 spectrophotometer (Thermo Scientific). One microgram of total RNA was converted to cDNA using RevertAid First Strand Synthesis Kit (Thermo Scientific, K1621) and RT2 First Strand Kit (SABiosciences, cat #330401). The level of specific transcripts was assessed using RT2 Profiler PCR Array Human Fibrosis (SABiosciences, cat #PAHS-120) according to the manufacturer's protocols. Confirmation of RT2 Profiler PCR Array was performed by Q-PCR analysis using TaqMan Gene Expression Assays (Applied Biosystems, cat #4331182). All PCR reactions were performed using 7500 Real-Time PCR System (Applied Biosystems). RT2 Profiler PCR Array data were analyzed using RT2 Profiler Array Data Analysis Version 3.5 Software. RT-

RCR data were analyzed using comparative ΔΔCt method. If not specified, GAPDH used as endogenous reference.

### Statistical analysis

The proliferative activity at different conditions was analyzed using statistical Kaplan-Meier method. Log-rank test was performed to determine the difference between two groups. The one-way analysis of variance (ANOVA) was used to determine whether there are significant differences between the means of three or more groups (GraphPad Prism version 6 for Windows). Results are expressed as mean ± S.E. Values considered to be statistically different when p < 0.05.
